# Predictive vs. Flow-Derived Haemodynamic Monitoring in Major Abdominal Surgery: Associations with Intraoperative Hypotension and Postoperative Outcomes

**DOI:** 10.3390/medsci14020210

**Published:** 2026-04-24

**Authors:** Alejandro Martín-Arrabal, Francisco M. Peinado, Miguel A. Arrabal-Polo, Antonio J. Gálvez-Muñoz, Tomás Saz-Terrado, María M. Olvera-García, María S. Serrano-Atero, Simón López-Soto, Mariana F. Fernández

**Affiliations:** 1 Department of Anesthesiology, San Cecilio Clinical University Hospital, 18007 Granada, Spain; alejandro.martin.arrabal.sspa@juntadeandalucia.es (A.M.-A.); tomas.saz.sspa@juntadeandalucia.es (T.S.-T.); mariam.olvera.sspa@juntadeandalucia.es (M.M.O.-G.); ms.serrano.sspa@juntadeandalucia.es (M.S.S.-A.); simon.lopez.sspa@juntadeandalucia.es (S.L.-S.); 2 Department of Surgical Specialties, Biochemical and Immunology, Faculty of Medicine, University of Málaga, 29071 Málaga, Spain; peinadofm@uma.es; 3 Medical Oncology Clinical Management Unit, Málaga Biomedical Research Institute (IBIMA)-CIMES-UMA, Virgen de la Victoria University Hospital, 29010 Málaga, Spain; 4 Biosanitary Research Institute (ibs.GRANADA), 18016 Granada, Spain; mangel.arrabal.sspa@juntadeandalucia.es (M.A.A.-P.); ajgalvez@ugr.es (A.J.G.-M.); 5 Centre for Biomedical Research (CIBM), University of Granada, 18016 Granada, Spain; 6 Consortium for Biomedical Research in Epidemiology and Public Health (CIBERESP), 28029 Madrid, Spain

**Keywords:** intraoperative hypotension, haemodynamic monitoring, hypotension prediction index, Vigileo/FloTrac system, postoperative outcomes

## Abstract

**Introduction:** Intraoperative hypotension (IOH) is a frequent manifestation of haemodynamic instability during general anaesthesia. Advances in arterial waveform analysis have led to two distinct monitoring strategies: flow-derived platforms and predictive algorithms designed to anticipate hypotension. However, prospective comparisons and their associations with IOH and postoperative outcomes remain limited. The objective was to compare predictive haemodynamic monitoring using the Hypotension Prediction Index (HPI) with flow-derived monitoring using the Vigileo/FloTrac system and to evaluate their associations with IOH and postoperative outcomes. **Methods:** In this single-center prospective observational study, 101 adults undergoing elective major abdominal surgery under general anaesthesia were monitored using either the HPI system (*n* = 49) or the Vigileo/FloTrac system (*n* = 52). Primary outcomes were cumulative duration and frequency of IOH (mean arterial pressure < 65 mmHg). Secondary outcomes included postoperative complications, organ injury biomarkers (troponin, creatinine, eGFR), and hospital length of stay. Multivariable regression models adjusted for predefined confounders were used to estimate associations. **Results:** Vigileo/FloTrac monitoring, compared with HPI, was independently associated with a greater cumulative duration of IOH (adjusted β = 1.66; 95% CI, 0.63–2.72) and a higher number of hypotensive episodes (adjusted β = 0.53; 95% CI, 0.10–0.95). Monitoring strategy was not associated with surgical site, respiratory, or neurological complications. However, Vigileo/FloTrac monitoring was associated with higher odds of vascular complications (adjusted OR = 4.36; 95% CI, 1.13–20.41). No significant associations were observed between monitoring strategy and postoperative organ injury biomarkers or length of hospital stay. **Conclusions:** Predictive haemodynamic monitoring using the HPI system was associated with lower IOH burden compared with the Vigileo/FloTrac system. However, these differences were not consistently accompanied by improvements in postoperative outcomes. Haemodynamic optimisation should be considered as one component within a broader, integrated perioperative management strategy. Further large-scale, multicenter prospective studies are warranted to clarify its impact on patient-centered outcomes.

## 1. Introduction

More than 300 million surgical procedures are performed worldwide each year [1], and a substantial proportion of patients who undergo surgery continue to experience intra- and postoperative complications [2], despite ongoing advances in perioperative care. Intraoperative haemodynamic management plays a pivotal role in perioperative medicine, as it is essential to maintain adequate blood pressure and circulatory stability in order to preserve tissue perfusion and prevent organ dysfunction [3,4]. Haemodynamic instability is quite common during general anaesthesia and has been associated with perioperative complications and adverse clinical outcomes [5,6,7,8].

Intraoperative hypotension (IOH) is one of the most frequent manifestations of haemodynamic instability during general anaesthesia [9,10]. Although its definition varies across studies, most agree that a mean arterial pressure (MAP) below 65 mmHg is the threshold most strongly associated with an increased risk of adverse postoperative outcomes [9], including acute kidney injury [11], myocardial injury [12], neurological complications [13], and an increased risk of mortality [6] in both cardiac and non-cardiac surgery. Furthermore, there is growing evidence to suggest that not only the occurrence of hypotensive events/episodes, but also their severity and cumulative duration, may influence postoperative recovery [14]. However, despite the knowledge and growing awareness of the clinical impact of IOH, haemodynamic management during surgery often remains predominantly reactive, with treatment only initiated once hypotension has already occurred. These conditions have driven increasing interest in more advanced monitoring strategies that allow for the anticipation of haemodynamic instability, supporting a more proactive approach to perioperative management [15,16].

Traditional techniques for haemodynamic monitoring, such as pulmonary artery catheterisation (Swan-Ganz catheter) or oesophageal Doppler monitoring, provide detailed physiological information but are limited by their own technical complexity and procedural risks [17]. Arterial waveform-based technologies have emerged as less invasive alternatives, enabling a continuous haemodynamic assessment during non-cardiac surgery [10,18,19]. However, the most recent advances in perioperative haemodynamic monitoring have aimed to improve cardiovascular assessment whilst reducing invasiveness [18,20]. These approaches include distinct conceptual strategies, such as flow-derived monitoring platforms for example, the Vigileo/FloTrac system, which provides a continuous estimate of cardiac output, among other dynamic haemodynamic variables, as well as supporting fluid and vasoactive management [21,22]. Meanwhile, predictive algorithm-based platforms, such as the Hypotension Prediction Index (HPI), apply machine-learning analysis to anticipate hypotensive events before they occur [23]. The HPI system applies machine-learning algorithms to the arterial pressure waveform to generate a unitless index (0–100) reflecting the probability of imminent hypotension, whilst the Vigileo/FloTrac system derives continuous cardiac output and dynamic haemodynamic variables from pulse contour analysis, integrating waveform characteristics and patient-specific demographic data to account for vascular tone.

Flow-guided monitoring strategies have been associated with improved haemodynamic optimisation in specific contexts, although the available evidence remains heterogeneous across surgical populations [20,23,24]. The scientific literature also suggests that HPI-guided haemodynamic management could reduce the IOH burden, although its impact on postoperative outcomes remains uncertain [23,25,26,27,28]. Despite this evidence, direct prospective comparisons evaluating the clinical efficacy of flow-derived versus predictive monitoring strategies remain limited, and most of the available studies come from retrospective and highly heterogeneous cohorts, preventing the generalization of current evidence.

Furthermore, beyond intraoperative blood pressure parameters, the impact of these monitoring strategies on perioperative complications and biomarkers of organ damage has not yet been comprehensively described in routine surgical practice. Additionally, perioperative haemodynamic responses are influenced by multiple patient- and procedure-related factors [29], highlighting the need to better identify the variables associated with hypotension burden and postoperative outcomes.

In this context, the main objective of this study was to compare two conceptually distinct arterial haemodynamic monitoring strategies, the HPI system and the Vigileo/FloTrac system, in a cohort of adult patients undergoing major non-cardiac abdominal surgery. The objective was to evaluate the association between the monitoring strategy adopted and the IOH burden. As secondary objectives, the associations between the monitoring strategy and perioperative complications, biomarkers of postoperative organ injury, and length of hospital stay (postoperative recovery) were also analyzed. Exploratory analyses were additionally carried out to identify independent clinical and intraoperative variables associated with the IOH burden and postoperative outcomes.

## 2. Material and Methods

### 2.1. Study Design and Population

A single-center prospective observational comparative study was conducted at University Hospital San Cecilio (Granada, Spain), involving 101 adult patients who underwent non-cardiac abdominal surgery between January 2023 and January 2024. All procedures were scheduled surgeries, either open or laparoscopic surgeries, performed under general anaesthesia with controlled mechanical ventilation. Inclusion criteria also included operations requiring invasive arterial monitoring and patients with a physical status of I-III according to the American Society of Anesthesiologists (ASA). Exclusion criteria comprised severe chronic kidney disease with an estimated glomerular filtration rate (eGFR) ≤ 30 mL/min/1.73 m^2^, dependence on dialysis, haemodynamic instability, emergency surgery, cardiac or vascular procedures, regional anaesthesia as the primary technique, spontaneous ventilation, and pregnancy.

All patients received oral and written information about the study objectives and provided written informed consent prior to inclusion. The study was approved by the Provincial Ethics Committee of Granada (TFM-MVVH-2022, approval on 23 December 2022), and conducted in accordance with the principles of the Declaration of Helsinki and current Spanish biomedical research regulations (Law 14/2007 on Biomedical Research, Royal Decree 1716/2011, and Organic Law 3/2018 on Data Protection and Digital Rights).

To ensure a fair comparison of the selected monitoring strategies, patients were randomly assigned to either the HPI system or the Vigileo/FloTrac system, following a predefined sequential inclusion strategy designed to ensure a similar distribution across both groups in terms of age, sex and type of surgery. The investigators did not protocolize or modify any therapeutic interventions; no standardized haemodynamic management protocol was implemented; and all intraoperative management decisions, including fluid administration and vasoactive support, were left to the discretion of the anaesthetist in charge.

### 2.2. Sample Size

Sample size was based on the previous studies [30,31], in which the primary outcome measure was a 75% reduction in hypotension, in terms of both intensity and duration, with a time-weighted average estimated IOH of 0.5 mm Hg, and a mean difference between groups of 0.38 mm Hg. With a sample size of 60 subjects, the researchers achieved 80% power to detect an effect size of at least 0.74, with an estimated difference of 0.38 between subjects receiving different haemodynamic treatments, using Student’s *t*-test with a two-tailed significance level of 0.05. In our study, the variability of the primary variable was also considered, and an adjustment for losses or exclusions was applied, increasing that estimated sample size to compensate for potential dropouts, incomplete data or technical problems with monitoring. The sample size also took into account the average annual number of patients undergoing major abdominal surgery at the HUCSC. All consecutive eligible patients were included, resulting in a final sample of 101 patients.

### 2.3. Perioperative Management and Arterial Monitoring

All patients underwent general anaesthesia according to routine institutional clinical practice. Standard intraoperative monitoring included five-lead electrocardiography, heart rate, invasive arterial blood pressure, peripheral oxygen saturation, temperature monitoring, bispectral index, and neuromuscular blockade assessment using train-of-four. Invasive arterial pressure was measured through a radial arterial catheter, preferentially placed in the left radial artery.

Arterial haemodynamic monitoring was carried out in accordance with standard clinical practice, including fluid therapy, the administration of vasoactive drug and ventilatory adjustments, and was guided by the anaesthesiologist in charge based on the available monitoring data. Both haemodynamic monitoring systems, the HPI system or the Vigileo/FloTrac system, were initiated prior to induction of anaesthesia, maintained for 5–10 min following induction, and recorded continuously throughout the surgical procedure and during the immediate postoperative recovery period.

### 2.4. Outcomes

#### 2.4.1. Primary Outcome

The primary outcome was IOH burden, including the total duration of hypotension (min) and number of hypotensive events during surgery. Consecutive hypotensive episodes separated by less than one minute were considered part of the same event. IOH was defined as a MAP below 65 mmHg for at least one minute, consistent with thresholds commonly used in the previous literature [8,30,31].

#### 2.4.2. Secondary Outcomes

Postoperative complications, postoperative organ injury biomarkers, and postoperative recovery, were assessed within the first 24 h and up to 7 days after surgery. Postoperative complications included surgical site, vascular, respiratory, and neurological complications. Surgical site complications were defined as the occurrence of surgical wound infection, suture dehiscence, or need for surgical reintervention. Vascular complications included myocardial injury, clinically significant arrhythmias, and thromboembolic events. Respiratory complications comprised pneumonia, respiratory failure, or the need for ventilatory support. Neurological alterations referred to the occurrence of delirium, stroke, or postoperative cognitive impairment, as documented in clinical records. Postoperative organ injury was assessed by circulating biomarkers related to cardiac and renal function, such as troponin levels, serum creatinine, and eGFR, obtained from routine hospital laboratory measurements. Postoperative recovery was evaluated through hospital length of stay (days), calculated from the day of surgery until hospital discharge.

### 2.5. Covariates and Potential Confounders

All participants underwent anthropometric and clinical assessment, recording their height and weight for calculation of their body mass index (BMI). Sociodemographic and clinical variables were prospectively collected from routine medical records, including age, sex, diabetes, preoperative hypertension, chronic kidney disease, cardiovascular history, use of angiotensin-converting enzyme (ACE) inhibitors or angiotensin receptor blockers (ARBs), beta-blockers, baseline haemoglobin levels, and ASA physical status. Perioperative variables included type of surgery (distal pancreatectomy, total pancreatectomy, pancreaticoduodenectomy, liver resection, partial gastrectomy, total gastrectomy, intestinal surgery, oesophageal surgery, and other procedures), surgical approach (open or laparoscopic), type of general anaesthesia (desflurane, sevoflurane, or propofol), epidural catheter use, duration of surgery, and estimated intraoperative bleeding (low, moderate, or severe). Type of surgery was categorized into three major groups (pancreatic, hepatic, and gastric surgery) to ensure model parsimony and reduce the risk of overfitting.

Potential confounders were identified a priori based on clinical relevance and causal assumptions, supported by directed acyclic graphs (DAGs) (Figure S1). These variables included age, sex, ASA physical status, type of surgery, surgical approach, duration of surgery, and use of epidural catheter.

### 2.6. Statistical Analysis

Descriptive analyses were performed to summarize baseline, intraoperative, and postoperative characteristics of the study population. Continuous variables were expressed as mean ± standard deviation (SD), while categorical variables were reported as frequencies (percentages). The distribution of continuous variables was assessed qualitatively using boxplots, histograms, and Q-Q plots, and quantitatively using the Shapiro–Wilk test. Continuous variables with skewed distributions were transformed to standardize their distributions and minimize the influence of extreme values. Specifically, duration of IOH (min) and number of hypotensive events were square-root transformed, whereas troponin levels, serum creatinine, and hospital length of stay (days) were natural log-transformed.

Bivariate analyses were conducted to compare baseline, intraoperative, and postoperative characteristics between both arterial monitoring strategies. Continuous variables were analyzed using Student’s *t*-test, Mann–Whitney U test, or Kruskal–Wallis test, as appropriate, while categorical variables were compared using the Chi-square test or Fisher’s exact test. Statistical significance was defined as a two-sided *p*-value < 0.05.

#### 2.6.1. Association Between Arterial Monitoring Strategy and Intraoperative Hypotension

Univariable and multivariable linear regression models were conducted to evaluate the association between arterial monitoring strategy and the IOH burden. Separate models were performed for each IOH component, (total duration of IOH and the number of hypotensive events). Unadjusted models were first estimated to assess crude associations. Subsequently, multivariable models were adjusted for a predefined set of potential confounders. Effect estimates were reported as regression coefficients (β) with 95% confidence intervals (95% CI) and *p*-values, with CI derived using bias-corrected and accelerated (BCa) bootstrap resampling (10,000 iterations) to obtain robust effect estimates.

#### 2.6.2. Association Between Arterial Monitoring Strategy and Secondary Outcomes

Univariable and multivariable logistic regression models were used to evaluate the association between arterial monitoring strategy and postoperative complications, which were analyzed as binary outcomes (presence vs. absence). Linear regression models were applied to postoperative organ injury biomarkers and hospital length of stay (days). For both analyses, unadjusted models were first estimated to assess crude associations, followed by models adjusted for a predefined set of potential confounders. An additional exploratory model further incorporated total duration of IOH into the adjustment set to evaluate its influence on the observed associations. Effect estimates were reported as odds ratios (ORs) or regression coefficients (β), as appropriate, together with 95% CI, estimated using BCa bootstrap resampling, and *p*-values.

Estimated intraoperative bleeding (categorized as low, moderate, or severe) was additionally included in the multivariable models, as a sensitivity analysis, to assess whether bleeding modified the associations between the monitoring strategy and the study results.

#### 2.6.3. Additional and Stratified Analyses

Additional multivariable regression models were constructed to assess factors independently associated with the study outcomes. Linear regression models were applied to continuous outcomes, whereas logistic regression models were used for binary clinical outcomes. Candidate variables were selected a priori based on clinical relevance, prior evidence, and DAGs, including demographic characteristics, comorbidities, surgical factors, anaesthetic management, and haemodynamic variables. Exploratory interaction terms between arterial monitoring strategy, type of surgery, and surgical approach were examined to explore potential effect modification, guided by clinical plausibility. Model performance was evaluated using adjusted R^2^ for linear models and the area under the receiver operating characteristic curve (ROC AUC) to evaluate discrimination of logistic regression models. Effect estimates were reported as regression coefficients (β) or ORs, as appropriate, together with 95% CI, estimated using BCa bootstrap resampling, and *p*-values.

Descriptive stratified analyses were conducted to explore potential heterogeneity across clinically relevant subgroups according to surgical approach (open vs. laparoscopic), type of surgery (pancreatic vs. hepatic vs. gastric), type of anaesthesia (sevoflurane, propofol, or desflurane), and epidural catheter use (yes vs. no), evaluating the distribution of baseline, intraoperative, and postoperative characteristics across arterial monitoring strategies.

#### 2.6.4. Software

SPSS v28.0.1.0 (IBM, Chicago, IL, USA) and the R statistical computing environment v4.4.1 (http://www.r-project.org/, accessed on 19 April 2026) were used for data analyses.

## 3. Results

### 3.1. Characteristics of the Study Population

Baseline, intraoperative, and postoperative characteristics of the study population are summarized in Table 1. The cohort of 101 patients undergoing major non-cardiac abdominal surgery was randomly allocated to either the HPI monitoring system (*n* = 49) or the Vigileo/FloTrac system (*n* = 52), with both groups demonstrating comparable baseline characteristics. The mean (SD) age of the population was 64.7 (12.3) years, and two-thirds of the population were men. The prevalence of hypertension and diabetes was 51.5% and 25.7%, respectively, with no significant differences between the groups. Chronic treatment with ACE inhibitors or ARBs was reported in 43.6% of patients, and use of beta-blockers in 18.8%. Levels of certain laboratory parameters, such as haemoglobin [13.2 (1.3) g/dL], creatinine [0.8 (0.2) mg/dL], and eGFR [89.3 (17.7) mL/min/1.73 m^2^], were also similar between the groups. Most patients were classified as ASA II or III (93.1%).

Intraoperative characteristics were also comparable between patients in the different monitoring strategies. There were no significant differences in the distribution of surgical approach, type of general anaesthesia, or use of epidural catheters; however, the estimated intraoperative bleeding varied between the groups, with a higher proportion of moderate bleeding observed in patients monitored with Vigileo/FloTrac (low:moderate:severe, 35:8:6 vs. 29:21:2). Furthermore, the HPI group experienced fewer intraoperative hypotensive events [3.6 (4.4) vs. 5.9 (5.0)] and a shorter cumulative duration of IOH [17.0 (20.2) vs. 34.0 (29.9) minutes] compared with the Vigileo/FloTrac group (Table 1).

Among the postoperative variables, there was a trend towards an increase in vascular complications in the Vigileo/FloTrac group (26.9% vs. 10.2%), whilst no significant differences were observed in respiratory or neurological complications, nor in the length of hospital stay between the two groups.

Stratified descriptive analyses according to surgical approach, type of surgery, type of anaesthesia, and epidural catheter use are presented in the Supplementary Tables S1–S4. In open surgery, moderate intraoperative bleeding was more frequent in the Vigileo/FloTrac group, whilst in laparoscopic procedures a higher IOH burden was observed, both in terms of duration and number of events, also in the Vigileo/FloTrac group. Similarly, patients undergoing hepatic surgery and those receiving desflurane anaesthesia showed a higher IOH burden when monitored with Vigileo/FloTrac. In gastric surgeries, hypertension and chronic treatment with ACE inhibitors or ARBs were more prevalent in the Vigileo/FloTrac group. A higher IOH burden was also observed among Vigileo/FloTrac patients using an epidural catheter.

### 3.2. Association Between Arterial Monitoring Strategy and IOH

Arterial monitoring with the Vigileo/FloTrac system was associated with a greater cumulative duration of IOH compared with the HPI system (adjusted β = 1.66; 95% CI: 0.63–2.72). Similar results were observed in univariable models (β = 1.80; 95% CI: 0.73–2.92). Patients monitored with Vigileo/FloTrac experienced a higher number of intraoperative hypotensive events than those monitored with the HPI system (adjusted β = 0.53; 95% CI: 0.10–0.95), with consistent findings in unadjusted analyses (β = 0.60; 95% CI: 0.16–1.07) (Table 2).

### 3.3. Association Between Arterial Monitoring Strategy and Secondary Outcomes

No significant associations were found between arterial monitoring strategy and surgical site complications. Patients monitored with Vigileo/FloTrac showed higher odds of vascular complications compared with the HPI system (adjusted OR = 4.36; 95% CI: 1.13–20.41), with comparable results after additional adjustment for IOH duration in exploratory models (OR = 5.26; 95% CI: 1.22–29.20). No associations were observed for respiratory complications or neurological complications (Table 3).

Regarding postoperative organ injury biomarkers and recovery, monitoring strategy was not associated with troponin concentrations, serum creatinine, eGFR, or hospital length of stay. Additional adjustment for IOH duration yielded similar results (Table 4). Similarly, adjustment for estimated intraoperative bleeding did not materially modify the magnitude, direction, or statistical significance of the observed associations.

### 3.4. Factors Associated with Intraoperative Hypotension Burden

In the exploratory multivariable models including perioperative variables, several factors were independently associated with IOH burden (Table S5). Use of the Vigileo/FloTrac system remained associated with a longer cumulative duration of IOH (β = 0.45; 95% CI: 0.01–0.89) and with a higher number of hypotensive events (β = 0.57; 95% CI: 0.12–1.03). Age was independently associated with prolonged hypotension duration (β = 0.02; 95% CI: <0.01–0.04), whereas being a female patient was associated with shorter hypotension time (β = −0.87; 95% CI: −1.32 to −0.41).

### 3.5. Factors Associated with Postoperative Complications, Postoperative Organ Injury Biomarkers, and Recovery Outcomes

#### 3.5.1. Postoperative Complications

The use of the Vigileo/FloTrac system was associated with vascular complications (OR = 7.42; 95% CI: 1.66–45.30). Longer surgical duration (OR = 1.01; 95% CI: 1.00–1.02) and severe intraoperative bleeding (OR = 22.94; 95% CI: 1.97–340) were associated with higher odds of respiratory complications, whereas chronic pulmonary disease was associated with lower odds (OR = 0.15; 95% CI: 0.02–0.82). Severe intraoperative bleeding was also associated with neurological complications (OR = 12.22; 95% CI: 1.62–129). The wide confidence intervals for severe intraoperative bleeding were consistent with the limited number of events for these outcomes (Table S6A).

#### 3.5.2. Postoperative Organ Injury Biomarkers

Monitoring with the Vigileo/FloTrac system was associated with lower postoperative troponin concentrations (β = −1.48; 95% CI: −2.54 to −0.55), whilst a history of vascular disease was associated with higher troponin levels (β = 0.94; 95% CI: 0.37–1.57). Compared with pancreatic surgery, liver surgery (β = −1.02; 95% CI: −2.07 to −0.06) and gastrointestinal/other procedures (β = −1.46; 95% CI: −2.51 to −0.58) were also associated with lower troponin concentrations. Conversely, the interaction between monitoring strategy and gastrointestinal/other procedures was associated with higher troponin levels (β = 1.52; 95% CI: 0.25–2.86) (Table S6B).

Higher BMI was associated with increased postoperative creatinine concentrations (β = 0.03; 95% CI: 0.01–0.04). Higher BMI (β = −1.32; 95% CI: −2.52 to −0.19) and older age (β = −1.03; 95% CI: −1.42 to −0.61) were associated with reduced eGFR. Longer surgical duration (β = 0.003; 95% CI: 0.002–0.004) and history of vascular disease (β = 0.99; 95% CI: 0.44–2.04) were associated with higher eGFR. The interaction between monitoring strategy and gastrointestinal/other procedures was associated with reduced eGFR (β = 2.25; 95% CI: 0.77–5.58) (Table S6B).

#### 3.5.3. Postoperative Recovery

Longer surgical duration was associated with longer hospital length of stay (β = 0.003; 95% CI: 0.002–0.004). Use of sevoflurane (β = −0.45; 95% CI: −0.74 to –0.16) and propofol (β = −0.39; 95% CI: −0.67 to −0.14) was associated with shorter hospital stay, whereas no consistent associations were observed for sex or surgical type (Table S6B).

## 4. Discussion

In this prospective observational cohort study of adults undergoing major non-cardiac abdominal surgery, two conceptually distinct arterial haemodynamic monitoring strategies were compared: a predictive machine-learning-based approach (HPI) and a pulse-contour flow-derived platform (Vigileo/FloTrac), evaluating their associations with IOH burden as well as postoperative outcomes. HPI monitoring was associated with a lower IOH burden, including fewer hypotensive events and a shorter cumulative duration of these episodes. However, postoperative outcomes were comparable between the two monitoring systems, although a higher frequency of vascular complications was observed among patients monitored with Vigileo/FloTrac. No consistent differences were observed in biomarkers of organ damage or in length of hospital stay between the two study groups.

### 4.1. Association Between Monitoring Strategies and Intraoperative Hypotension

The anticipatory design of predictive haemodynamic monitoring systems may partly explain the lower IOH burden observed in patients using HPI monitoring, whereas the Vigileo/FloTrac system operates within a more reactive management model [32]. These conceptual differences provide a plausible mechanistic basis for the divergent intraoperative haemodynamic profiles between the groups. Previous studies suggest that HPI can predict impending hypotension and may reduce IOH when integrated into haemodynamic decision-making [10,23,26], with one meta-analysis reporting reductions in both duration and severity [23]. However, the available evidence remains heterogeneous, with variability in treatment algorithms, clinician experience, perioperative context, and hypotension definitions across studies [6,9,24,28,29,31,33]. Evidence specifically evaluating the impact of the Vigileo/FloTrac system on IOH, as well as direct prospective comparisons between predictive and flow-derived approaches, remains limited. From a physiological perspective, predictive monitoring may shift haemodynamic management from correction of established hypotension to prevention of impending instability, enabling earlier identification of haemodynamic vulnerability and intervention before sustained reductions in perfusion pressure occur. In this context, our findings of reduced IOH with HPI monitoring suggest that monitoring strategies incorporating predictive algorithms may facilitate more proactive haemodynamic management and support the idea that predictive monitoring can improve intraoperative haemodynamic control compared to reactive, flow-derived monitoring in routine clinical practice.

### 4.2. Association Between Monitoring Strategies and Postoperative Outcomes

#### 4.2.1. Postoperative Complications

Substantial evidence has reported associations between IOH burden and postoperative complications, including surgical site morbidity [9,14], acute kidney injury [8,34], respiratory and cardiovascular events [8,12], neurological complications [33,35], and mortality [6,36], reflecting the adverse systemic consequences of impaired tissue perfusion during surgery [6,7,14]. However, it remains uncertain whether reducing IOH through advanced haemodynamic monitoring translates into improved clinical outcomes.

While advanced haemodynamic monitoring has been associated with reductions in IOH, most of the available evidence on postoperative outcomes comes from HPI-guided strategies and remains heterogeneous [24,26,31]. Some studies suggest possible reductions in cardiovascular, neurological, and mortality outcomes [37,38], while a recent randomized clinical trial in abdominal surgery reported no decrease in acute kidney injury or mortality [24]. Evidence specifically addressing the impact of flow-derived platforms, such as Vigileo/FloTrac, on postoperative complications is also scarce. Consistent with Khwannimit, Sathaporn [25], we observed no significant associations between monitoring strategies and surgical site, respiratory, or neurological postoperative complications, indicating that differences in intraoperative haemodynamic management are insufficient to influence postoperative outcomes. In our study, additional adjustment for IOH did not alter the associations found, suggesting that IOH may not be the dominant causal or mediating mechanism linking monitoring strategy to postoperative outcomes. Only a higher frequency of vascular complications was observed among patients monitored with Vigileo/FloTrac; however, the wide confidence intervals of this association warrant a cautious interpretation of these results.

#### 4.2.2. Organ Injury Biomarkers

IOH has also been consistently associated with postoperative organ injury, particularly myocardial and renal dysfunction [39,40], likely reflecting transient mismatches between oxygen supply and demand and subsequent reperfusion-related cellular stress [11,12,31]. However, direct evidence linking specific haemodynamic monitoring strategies to postoperative organ injury biomarkers remains scarce [41], and comparative data between predictive and flow-derived platforms are limited. In our study, the monitoring strategy was not associated with postoperative troponin concentrations or renal function biomarkers. Although predictive haemodynamic algorithms have been associated with reductions in oxidative stress and markers of neuronal injury [41], the magnitude of IOH in our cohort may have been insufficient to induce measurable damage, and the early biomarkers collected may incompletely capture subtle perfusion differences.

#### 4.2.3. Postoperative Recovery

Length of hospital stay is a clinically relevant marker of postoperative recovery, influenced not only by surgical complexity and perioperative complications but also by baseline patient status and institutional care pathways [27]. Evidence regarding the impact of intraoperative haemodynamic management on length of stay remains heterogeneous, with some studies reporting no reduction [24,42] and others suggesting shorter hospitalization following HPI-guided optimization [27]. In our cohort, the monitoring strategy was not associated with hospital length of stay, suggesting limited influence of intraoperative haemodynamic differences on recovery trajectories.

### 4.3. Factors Associated with Intraoperative Hypotension and Postoperative Outcomes

Exploratory multivariable analyses identified independent associations across three interrelated domains: monitoring strategy, patient vulnerability, and procedural complexity. Use of the Vigileo/FloTrac system remained independently associated with longer cumulative hypotension duration and a higher number of hypotensive events, consistent with the primary analyses and suggesting that monitoring paradigm may influence intraoperative blood pressure dynamics. Increasing age was associated with prolonged hypotension duration, consistent with age-related vascular stiffness and reduced physiological reserve [29], whereas female sex was associated with shorter hypotension duration, although underlying mechanisms remain uncertain, and may relate to sex-related differences in vascular regulation [43,44].

Stratified descriptive analyses suggested that the haemodynamic impact of monitoring strategy may vary according to procedural and anaesthetic context. The higher IOH burden observed with Vigileo/FloTrac was more pronounced in laparoscopic and hepatic procedures, during desflurane-based anaesthesia, and in patients receiving epidural analgesia. Although exploratory, these patterns indicate that monitoring effects may be amplified in settings characterized by haemodynamic instability.

Postoperative complications may also be primarily driven by procedure and patient-related factors. Thus, longer surgery duration was associated with vascular and respiratory complications in our study, suggesting procedural complexity, sustained inflammatory activation, and/or cumulative physiological stress [45]. Severe intraoperative bleeding was also associated with respiratory complications, consistent with haemodynamic instability and reduced pulmonary reserve [46]; however, when this qualitative variable was included in the adjusted multivariate model, the main found associations remained unchanged/unaffected. Conversely, chronic pulmonary disease was associated with lower odds of respiratory complications in multivariable analyses. This counterintuitive finding may reflect residual confounding, differences in perioperative management, or a limited number of events rather than a true protective effect. Vascular disease was strongly associated with higher postoperative troponin concentrations [47], underscoring the dominant influence of baseline cardiovascular vulnerability. The association between Vigileo/FloTrac use and vascular complications should be interpreted cautiously given the wide confidence intervals and the observational design of the study. Associations with surgical type and the interaction between monitoring strategy and surgical category further suggest that perioperative myocardial stress is strongly influenced by procedural context [48]. Epidural catheterisation was associated with lower odds of vascular complications, possibly related to altered haemodynamic responses to surgical stress [49].

Higher BMI and advanced age were associated with lower renal functional indices, consistent with reduced renal reserve [50], while longer surgical duration and vascular disease were also associated with changes in eGFR, suggesting cumulative physiological stress and baseline vascular health [51]. Length of hospital stay was primarily determined by surgical duration, whereas associations with anaesthetic technique possibly reflect care pathways rather than direct physiological effects.

Overall, our findings reinforce the concept that perioperative risk is inherently multidimensional, resulting from the interaction between patient susceptibility and procedural stress. Haemodynamic monitoring may modulate IOH, but represents only one component within the complex framework of perioperative care.

### 4.4. Strengths and Limitations

This study offers several strengths primarily stemming from its prospective design. The inclusion of patients undergoing major abdominal surgery also provides a real-world assessment of haemodynamic monitoring strategies in routine clinical practice. The direct comparison between two conceptually distinct monitoring paradigms, along with the integrated evaluation of intraoperative haemodynamic exposure, postoperative complications, biomarkers of organ damage, and recovery outcomes, offers a comprehensive evaluation of perioperative risk that extends beyond isolated physiological metrics. Multivariable adjustment for clinically relevant perioperative variables further supports the internal validity of the findings.

Our findings should, however, be interpreted with caution due to several limitations. Firstly, the observational design precludes causal inference, and residual confounding, including confounding by indication, cannot be ruled out. In addition, no standardized haemodynamic management protocol was implemented, as intraoperative management—including fluid administration and vasoactive support—was left to the discretion of the attending anaesthetist without predefined therapeutic algorithms. While this approach reflects real-world clinical practice, it may have introduced variability in treatment responses. The modest sample size may have limited the statistical power for outcomes with low incidence rates and contributed to the imprecision of some estimates, as reflected by some wide confidence intervals. In particular, the relatively low number of events for certain outcomes, such as vascular complications, resulted in wide confidence intervals, warranting cautious interpretation of these findings. Moreover, the inclusion of heterogeneous surgical procedures (pancreatic, hepatic, and gastric) may have diluted potential effects of the monitoring strategy and limited the ability to detect procedure-specific differences. Although additional adjustment for IOH did not materially change the observed associations, suggesting that IOH may not represent the dominant causal or mediating mechanism linking monitoring strategy to postoperative outcomes, the use of a fixed MAP threshold (<65 mmHg) may not adequately capture individual patient physiology, particularly in those with chronic hypertension. Furthermore, the fact that the study was conducted in a single hospital, together with unmeasured factors such as variability in intraoperative decision-making, limits the generalizability of the results. Finally, the relatively short follow-up period may have restricted the detection of delayed postoperative complications as well as longer-term outcomes.

## 5. Conclusions

In this prospective observational study, predictive haemodynamic monitoring using the HPI system was associated with a lower IOH burden compared with flow-derived monitoring using the Vigileo/FloTrac system in patients undergoing major abdominal surgery. However, these differences in haemodynamic management did not translate into consistent improvements in postoperative complications, organ injury biomarkers, or length of hospital stay, underscoring the multifactorial nature of perioperative outcomes. Overall, our findings suggest that although advanced haemodynamic monitoring may contribute to the modulation of intraoperative hypotension—and reductions in IOH are clinically relevant—it should be regarded as only one component within a broader, integrated perioperative management strategy. Further large-scale, multicenter prospective studies are warranted to better delineate the clinical impact of different haemodynamic monitoring approaches on patient-centered and longer-term outcomes.

## Figures and Tables

**Table 1:** Baseline, intraoperative and postoperative characteristics of the study population.

	Whole Population (*n* = 101)	HPI Monitoring (*n* = 49)	Vigileo/FloTrac Monitoring (*n* = 52)	*p*-Value *
**Baseline characteristics**				
Age (years)	64.7 ± 12.3	63.9 ± 13.0	65.5 ± 11.7	0.556
Sex (male:female)	67:34	33:16	34:18	0.835
BMI (kg/m^2^)	25.8 ± 4.2	26.1 ± 4.1	25.6 ± 4.4	0.731
Diabetes (%)	25.7	18.4	32.7	0.100
Hypertension (%)	51.5	51.0	51.9	0.928
ACE inhibitors or ARBs (%)	43.6	38.8	48.1	0.346
Beta-blockers (%)	18.8	20.4	17.3	0.690
Hemoglobin (g/dL)	13.2 ± 1.3	13.1 ± 2.0	13.4 ± 1.6	0.610
Creatinine (mg/dL)	0.8 ± 0.2	0.8 ± 0.2	0.8 ± 0.2	0.762
eGFR (mL/min/1.73 m^2^)	89.3 ± 17.7	90.0 ± 17.2	88.6 ± 18.2	0.804
Chronic renal insufficiency (%)	4.0	4.1	3.8	1.000
ASA (I:II:III)	7:37:57	4:21:24	3:16:33	0.339
Previous vascular events (%)	36.6	30.6	42.3	0.223
Previous vascular, respiratory, renal & neurological diseases (%)	75.2	67.3	82.7	0.074
**Intraoperative characteristics**				
Type of surgery (pancreatic:hepatic:gastric)	30:32:39	13:16:20	17:16:19	0.790
Surgical approach (open:laparoscopic)	62:39	28:21	34:18	0.395
General anaesthesia type (desflurane:sevoflurane:propofol)	62:31:31	11:4:7	27:14:13	0.665
Epidural catheter use (%)	54.5	55.1	53.8	0.899
Estimated bleeding (low:moderate:severe)	64:29:8	35:8:6	29:21:2	**0.017**
Surgical complications (%)	36.6	30.6	42.3	0.223
Hypotension events (n)	4.7 ± 4.8	3.6 ± 4.4	5.9 ± 5.0	**0.009**
Total hypotension time (minutes)	25.5 ± 26.7	17.0 ± 20.2	34.0 ± 29.9	**0.003**
**Postoperative characteristics**				
Vascular complications (%)	18.8	10.2	26.9	**0.032**
Respiratory complications (%)	25.7	22.4	28.8	0.462
Neurological alterations (%)	20.8	20.4	21.1	0.926
Hospital stay (days)	13.0 ± 10.0	12.8 ± 10.0	13.2 ± 10.0	0.757

Continuous variables are presented as mean ± standard deviation and categorical variables as percentage. BMI: Body Mass Index; ASA: American Society of Anesthesiologists physical status classification; HPI: Hypotension Prediction Index; ACE: Angiotensin-Converting Enzyme; ARB: Angiotensin Receptor Blocker; eGFR: estimated Glomerular Filtration Rate. * *p*-values were calculated using Student’s *t*-test or Mann–Whitney U test for continuous variables, and Chi-square test or Fisher’s exact test for categorical variables, as appropriate. Bold text indicates *p*-value < 0.05.

**Table 2:** Association between arterial monitoring strategy and intraoperative hypotension.

	Hypotension Time (Minutes)								Hypotension Events							
	β	95% CI		*p*-Value	β^1^	95% CI		*p*-Value	β	95% CI		*p*-Value	β^1^	95% CI		*p*-Value
Invasive arterial monitoring (Vigileo/FloTrac) ^a^	1.80	0.73	2.92	**0.002**	1.66	0.63	2.72	**0.002**	0.60	0.16	1.07	0.010	0.53	0.10	0.95	**0.018**

^a^ Reference category = HPI monitoring; CI: confidence interval. β: unadjusted coefficient; β^1^: adjusted coefficient for age, sex, ASA scale, type of surgery, surgical approach, duration of surgical intervention and use of epidural catheter. Hypotension time (minutes) and number of hypotensive events were square-root transformed to satisfy model assumptions. 95% confidence intervals were estimated using bias-corrected and accelerated (BCa) bootstrap resampling (10,000 iterations). Bold text indicates *p*-value < 0.05.

**Table 3:** Association between arterial monitoring strategy and postoperative complications.

	OR	95% CI	*p*-Value	OR^1^	95% CI	*p*-Value	OR^2^	95% CI	*p*-Value
**Postoperative complications**									
**Surgical site complications**	1.76	0.74–4.42	0.197	1.67	0.44–5.93	0.295	1.53	0.37–6.11	0.419
**Vascular complications**	3.71	1.17–14.21	**0.035**	4.36	1.13–20.41	**0.042**	5.26	1.22–29.20	**0.037**
**Respiratory complications**	1.27	0.47–3.58	0.625	1.72	0.28–13.86	0.383	1.92	0.25–26.59	0.337
**Neurological complications**	1.00	0.32–3.10	1.000	0.82	0.12–4.80	0.739	0.91	0.10–8.14	0.917

Reference category: HPI monitoring; estimates represent the association of Vigileo/FloTrac monitoring relative to HPI. CI: confidence interval. OR: unadjusted odds ratio; OR^1^: adjusted for age, sex, ASA scale, type of surgery, surgical approach, duration of surgical intervention and use of epidural catheter. OR^2^: additionally adjusted for hypotension time (exploratory model). 95% confidence intervals were estimated using bias-corrected and accelerated (BCa) bootstrap resampling (10,000 iterations). Bold text indicates *p*-value < 0.05.

**Table 4:** Association between monitoring strategy and postoperative organ injury biomarkers and postoperative recovery.

	β	95% CI	*p*-Value	β^1^	95% CI	*p*-Value	β^2^	95% CI	*p*-Value
**Cardiac injury**									
**Troponins**	−0.31	−0.91–0.27	0.300	−0.43	−1.01–0.12	0.140	−0.42	−1.01–0.18	0.175
**Renal function**									
**Creatinine**	0.07	−0.08–0.22	0.367	0.05	−0.10–0.20	0.524	0.05	−0.11–0.21	0.534
**eGFR**	−5.45	−15.75–5.05	0.298	−2.29	−10.99–6.40	0.612	−2.89	−11.91–6.71	0.547
**Postoperative recovery**									
**Hospital length of stay (days)**	0.10	−0.20–0.38	0.510	0.10	−0.13–0.34	0.401	0.19	−0.07–0.43	0.146

Reference category: HPI monitoring, estimates represent the association of Vigileo/FloTrac monitoring relative to HPI. CI: confidence interval; eGFR: estimated Glomerular Filtration Rate. β: unadjusted coefficient; β^1^: adjusted for age, sex, ASA scale, type of surgery, surgical approach, duration of surgical intervention and use of epidural catheter. β^2^: additionally adjusted for hypotension time (exploratory model). Troponins, creatinine and hospital length of stay were log-transformed. 95% confidence intervals were estimated using bias-corrected and accelerated (BCa) bootstrap resampling (10,000 iterations).

## Data Availability

The data presented in this study are available on request from the corresponding author (clinical data are not publicly available due to ethical and privacy restrictions).

## Supplementary Materials

The following supporting information can be downloaded at: https://www.mdpi.com/article/10.3390/medsci14020210/s1, Table S1: Baseline, intraoperative and postoperative characteristics stratified by surgical approach (open vs. laparoscopic); Table S2: Baseline, intraoperative and postoperative characteristics stratified by surgery type (pancreatic, hepatic, gastric); Table S3: Baseline, intraoperative and postoperative characteristics stratified by type of anaesthesia (sevoflurane, propofol, desflurane); Table S4: Baseline, intraoperative and postoperative characteristics stratified by epidural catheter use (with vs. without epidural catheter); Table S5: Factors associated with intraoperative hypotension: multivariable linear regression models (*n* = 101); Table S6A: Factors associated with postoperative complications: multivariable logistic regression models (*n* = 101); Table S6B: Factors associated with postoperative organ injury biomarker and recovery outcomes: multivariable linear regression models (*n* = 101); Figure S1. Directed acyclic graph (DAG) illustrating assumed causal relationships between haemodynamic monitoring strategy, intraoperative hypotension, and postoperative outcomes.
